# Strategies to Position the Clinical Academic Nurse in University, Teaching and General Hospitals

**DOI:** 10.1111/jan.70571

**Published:** 2026-03-07

**Authors:** Suzan Henderikx, Maud Heinen, Hester Vermeulen, Catharina Jacoba Van Oostveen

**Affiliations:** ^1^ Máxima Medical Center Veldhoven Veldhoven the Netherlands; ^2^ IQ Health Science Department Radboud University Medical Center Nijmegen the Netherlands; ^3^ Amphia Research Centre Amphia Hospital Breda the Netherlands; ^4^ Erasmus School of Health Policy & Management (ESHPM), Section Health Services Management & Organisation (HSMO) Erasmus University Rotterdam Rotterdam the Netherlands

**Keywords:** clinical academic careers, clinical academic nurse position, clinical nursing practice, qualitative research

## Abstract

**Aim:**

Explore the perspectives of Clinical Academic Nurses and stakeholders on strategies for positioning Clinical Academic Nurses in Dutch hospitals.

**Design:**

A descriptive qualitative study.

**Methods:**

Semi‐structured interviews and focus groups were conducted with Clinical Academic Nurses and stakeholders from five hospitals involved in the positioning of Clinical Academic Nurses. Data was analysed using thematic analysis to identify strategies for positioning these nurses.

**Results:**

Four themes emerged: (1) ‘Supportive vision and culture’ is crucial for a shared vision and enables a culture for consistent support in the positioning, (2) A clear defined and strategic ‘Position of Clinical Academic Nurses’ is needed for uniform positioning, (3) ‘Research infrastructure’ describes the important supportive elements, and (4) ‘Leadership’ describes Clinical Academic Nurses' pioneering role in aligning research with organisational goals which strengthens their position.

**Conclusion:**

Positioning Clinical Academic Nurses in hospitals requires a vision, well‐defined positions, a research infrastructure, and leadership support. Long‐term strategic investments are needed to integrate research into clinical nursing practice and recognise Clinical Academic Nurses as strategic assets.

**Implications for Policy and Practice:**

Positioning Clinical Academic Nurses requires visionary leadership, institutional commitment and investment in research infrastructure. The Nurse Advisory Board should support this by aligning positioning, support and evaluation with strategic policies. Strategic hospital‐academic partnerships foster research, education, mentorship and grant support. Clinical Academic Nurses should set measurable goals, proactively align research with clinical priorities and increase visibility to advance nursing practice.

**Impact:**

This study identifies empirically grounded insights into strategies to position Clinical Academic Nurses and offers actionable insights for management, policymakers and Clinical Academic Nurses to strengthen knowledge infrastructure and improve patient care.

**Reporting Method:**

COREQ.

**Patient or Public Contribution:**

Limited patient and public involvement, focusing on feedback on preliminary results.

## What Does This Paper Contribute to the Wider Global Clinical Community?

- This study provides strategies for positioning CANs into hospital settings, highlighting the importance of a vision, a well‐defined position of CANs, a research infrastructure, and leadership of CANs.
- The findings offer actionable insights for hospital management policymakers and CANS themselves for positioning, ensuring integration of research into clinical practice.
- A strong position for CANs contributes to the advancement of evidence‐based nursing worldwide, improving healthcare innovation and patient outcomes.

## 1 Introduction

Clinical Academic Nurses (CANs), who combine clinical practice and academic work, are essential in ensuring the safety, quality and efficiency of patient care (Segrott et al. 2006; van Oostveen et al. 2017). Together with ward nurses, CANs initiate clinically relevant research questions, design studies to address these questions, and guide the implementation of the research findings into practice (Berthelsen and Koreska 2021). Additionally, CANs strengthen their colleagues' research skills by offering guidance in data collection, analysis, interpretation and the application of evidence in practice (Berthelsen and Koreska 2021). They systematically apply Evidence‐Based Practice (EBP) principles and implement clinical guidelines to improve everyday nursing practice (Allen et al. 2023). This is important for continuous learning to improve the quality and safety of nursing care. In addition, employing CANs with a PhD in hospitals can also offer financial benefits for hospitals, contributing to more effective patient care and cost savings in the long term (Elgaard Sorensen et al. 2019).

Despite the importance of the position of CANs, clinical academic positions for nurses remain limited. The significance of these positions is not yet fully understood and universally recognised among nursing colleagues, managers and physicians (van Oostveen et al. 2017; Berthelsen and Holge‐Hazelton 2018). Consequently, progress in expanding these positions is constrained, resulting not only in a scarcity of role models but also in missed opportunities to leverage the benefits CANs bring to healthcare. This underutilisation undermines care quality and wastes investments in the academic education and expertise of CANs. Nurses who do hold academic positions are often found in universities rather than in clinical settings (Elgaard Sorensen et al. 2019).

Without career opportunities in hospitals, CANs often transition into positions in policy departments, universities, or universities of applied sciences (Brant 2015). This shift can lead to a loss of valuable skills and knowledge that are crucial for maintaining the quality of direct patient care (van Oostveen et al. 2017). To ensure a strong knowledge infrastructure that upholds the standard of nursing care, the proper positioning and retention of CANs in clinical settings are vital. Insight into strategies for positioning CANs in clinical settings will aid CANs themselves, policymakers and managers in enhancing career pathways and empowering CANs to contribute to improving patient care.

## 2 Background

Nursing is a relatively young academic discipline worldwide. Although the UK and US have already made significant strides, the Netherlands has lagged behind due to an ineffective lobby for scientific nursing education (Berthelsen and Holge‐Hazelton 2018; Martini et al. 2021). Consequently, scientific work and quality improvement in nursing have long been undervalued by managers, physicians and nurses themselves, influenced by the persistent belief that nursing is practice‐based work that does not require academic preparation (Verhoeven et al. 2023; Rafferty 2018). This perception has slowed the development of advanced positions such as CANs (Rafferty 2018).

A strong knowledge infrastructure is essential for the scientific underpinning of nursing practice and to achieve healthcare organisational goals (Ridge 2021). CANs are positioned at the intersection of clinical practice and research, enabling them to strengthen the nursing knowledge infrastructure and promote evidence‐based practice (Ridge 2021). They contribute directly to organisational priorities by fostering a research‐oriented practice culture and implementing evidence‐based interventions including clinical pathways and guidelines (Berthelsen and Koreska 2021; Allen et al. 2023).

Enabling nurses to combine clinical and academic work requires structural and cultural support, including formal career pathways, a financial support infrastructure such as departmental budgets, access to research grants and protected research time (van Oostveen et al. 2017; Fredericks et al. 2025; Paterson and Strickland 2023). However, lack of clear organisational vision regarding clinical academic careers for nurses limits the support and leadership needed at all organisational levels to integrate research into nursing practice (van Oostveen et al. 2017; Paterson and Strickland 2023). Because many managers lack research expertise and are pressured to prioritise operational capacity due to workforce shortages, organisational support for CAN positions remains limited (Paterson and Strickland 2023; Aspinall et al. 2024). Moreover, the under‐appreciation of academic tasks within nursing teams, combined with peer pressure, often leads to negative attitudes towards CANs and discourages talented nurses considering a clinical academic career (van Oostveen et al. 2017). Without strong leadership that supports CAN positions throughout the organisation, CANs' visibility and impact remain limited (Berthelsen and Koreska 2021; Fredericks et al. 2025), which can lead them to leave clinical practice.

CANs need clearly defined positions, expectations and responsibilities that are recognised and supported by managers and the board of directors (Paterson and Strickland 2023; Holge‐Hazelton et al. 2016). This requires an infrastructure with structured job descriptions, employment contracts, salary scales and benefits to help them balance clinical and academic work effectively (Grant et al. 2022). The availability of credible nurse academic role models is crucial in shifting attitudes towards academic work in nursing, increasing the visibility and impact of nursing research on patient care (van Oostveen et al. 2017; Fredericks et al. 2025). Mentorship from peers and experienced CANs further aids in developing essential academic skills, building a stable academic identity and gaining confidence (Roddam et al. 2019). Yet most role models work in universities rather than hospitals, leaving CANs with limited support in areas such as grant writing. Because hospitals lack adequate financial support structures, CANs rely heavily on grants to combine clinical and academic work (van Oostveen et al. 2017). Without adequate guidance in grant writing, they struggle to secure funding for protected academic time and career development opportunities, such as PhD or postdoctoral training. Consequently, nearly half of nurses with PhDs discontinue their research careers (Al‐Nawafleh et al. 2013), representing a significant loss of expertise essential for high‐quality patient care and a costly underutilisation of talent (Lasater et al. 2021).

Although physicians are often willing to collaborate with nurses in research, they typically operate within a different research paradigm, often rooted in Positivism (Kuijper et al. 2024). This can result in sidelining of academic career development for nurses and an overemphasis on purely clinical research positions, such as research nurse roles. This dynamic can reinforce traditional hierarchies and role misinterpretation, where nursing is seen as subordinate to medicine (van Oostveen et al. 2017; Aspinall et al. 2024). This may inadvertently diminish the unique contributions of nursing research, such as its holistic perspective and patient‐centred outcomes (van Oostveen et al. 2017).

Many studies emphasise the challenges and obstacles involved in positioning CANs. However, some examples of successful positioning demonstrate their significant potential. Fredericks et al. outline strategies to enhance research engagement among nurses and allied health professionals, including building capacity, research infrastructure, career pathways and leadership positions (Fredericks et al. 2025). Although valuable, particularly in cardiovascular care, these strategies are broadly defined and not specific to CANs. Insights into specific, empirically grounded strategies for establishing the CAN position across hospital types and specialisms remain limited. Addressing this knowledge gap will inform the international conversation about CAN positioning and support stakeholders in developing career pathways that enable CANs to fully utilise their competencies to improve patient care.

## 3 The Study

### 3.1 Aim

This study explores the perspectives of CANs and other stakeholders regarding the strategies for positioning CANs in Dutch university hospitals, teaching hospitals and general hospitals.

## 4 Methods

### 4.1 Design

A general qualitative design using Thorne's interpretive description was applied to generate practically meaningful insights rather than abstract theory (Thorne 2025). This approach guided an iterative process between data collection and analysis, emphasising interpretation and contextual understanding in exploring strategies for establishing the CANs' position (Thorne 2025). Semi‐structured interviews were conducted with nurse educators, clinical care managers, nursing professors, clinical nurses, policymakers in nursing and nurse directors as well as other healthcare directors. One focus group was conducted with CANs in each of the hospitals. The Consolidated Criteria for Reporting Qualitative Research (COREQ) were used to report the study (Tong et al. 2007).

### 4.2 Study Setting

The study was conducted in five Dutch hospitals, including two University Medical Centres (UMCs), two teaching hospitals and one general hospital. They were selected for their hospital type diversity and because they are considered frontrunners in establishing the CAN's position. Participating hospitals employed approximately 30 CANs in UMCs, seven in teaching hospitals and four in general hospitals.

### 4.3 Participants and Recruitment

A purposive sampling approach was applied to recruit participants for the semi‐structured interviews and the focus groups in each participating centre. Eligible stakeholders included nurse educators, clinical care managers, assistant professors or associate professors in nursing, clinical nurses, policymakers in nursing, nurse directors or other healthcare directors involved in establishing the CANs' position. Eligible CANs were those combining clinical practice and research activities and had a master's degree (or in training), PhD or were PhD candidates. These groups were selected for their direct involvement in positioning or experiencing the CANs' position, which ensured both strategic and practice‐based perspectives. Maximum variation sampling was used to include diversity in roles, experience and academic background. Concerning the CANs, the group was heterogeneous with respect to academic degree (MSc or PhD) and years of experience. In each hospital, a designated contact person was instructed to invite eligible participants via email for the semi‐structured interviews or the focus groups. The invitation email described the study, emphasised voluntary participation and included contact details. Interested participants contacted the authors via email.

### 4.4 Data Collection

All participants received an email including the study's objectives and an invitation to join the interviews or focus groups. Interviews and focus groups were conducted using an interview guide grounded in literature on CANs, which was iteratively refined as data were collected (Allen et al. 2023; Fredericks et al. 2025; Paterson and Strickland 2023; Aspinall et al. 2024; Grant et al. 2022) (see Boxes 1 and 2).

BOX 1 **Interview prompts.**

**Table:** 

*What prompted the creation of the position of the CAN?* Topics: vision on nursing research *In what ways was the position of the CAN developed?* Topics: stakeholders, process, facilitators, barriers, leadership, theoretical framework *How is the position of the CAN embedded?* Topics: operational factors (organisational support, financial resources, time allocation), policy, facilitators, barriers *What are the career opportunities for CANs in your hospital?* Topics: opportunities for academic qualifications, developing research skills, academic identity, mentoring, infrastructure, research capacity, facilitators, barriers

BOX 2 **Focus group prompts.**

**Table:** 

*What are your current perspectives and experiences regarding the position of the CAN?* Topics: position within the hospital, transition from nursing to research, organisational support (job profile, job remuneration, naming of the role, stakeholders), financial resources, time allocation, responsibilities, accountability, impact on patient outcomes, visibility within the hospital, interprofessional collaboration, realisation within the team, work satisfaction, recognition *What is needed to further develop the position of the CAN?* Topics: role development, expectations, opportunities *What are the career opportunities for CANs in your hospital?* Topics: opportunities for academic qualifications, developing research skills, academic identity, mentoring, facilitators, barriers

Relevant documents (e.g., strategic policy plans, research agendas, organisational reports) were collected by SH via contact persons at each hospital to provide contextual background on CAN positioning and inform the focus group discussions. Additional documents were obtained from branch organisations, including the Collaboration Top clinical Hospitals (STZ), the Dutch Association of Hospitals (NVZ), the Dutch Federation of University medical centres (NFU), the Professional Association of Healthcare assistants and Nurses (V&VN) and the Dutch Alumni Association for Nurse Scientists (AlumniVW), to capture broader policy developments relevant to the CAN position.

All interviews and focus groups were conducted in Dutch, audiotaped with consent and introduced by SH with a brief explanation of the study. During and after each session, memos were written to capture non‐verbal and contextual information.

#### 4.4.1 Interviews

A pilot interview was conducted to validate the interview guide. Additionally, a mirrored interview, where two researchers critically assessed SHs questioning and interaction, was used to refine the technique. The interviews were scheduled based on participants' availability and conducted by SH via Microsoft Teams for logistical convenience. Probing and prompting techniques elicited deeper responses.

#### 4.4.2 Focus Groups

The focus groups were organised by the contact persons and conducted on‐site in each hospital. SH moderated the sessions, while a second researcher (MH, CvO or HvN) observed, clarified responses, and monitored engagement. The focus groups followed the five‐stage approach of Finch et al. to facilitate discussion, see Table 1 (Finch et al. 2003).

**TABLE 1:** Five stages for focus group researchers (Finch et al. 2003).

Stage 1	Set the scene and establish ground rules
Stage 2	Introduce the participants
Stage 3	Choose a neutral opening topic
Stage 4	Discuss the area you wish to research
Stage 5	Debriefing the participants

### 4.5 Data Analysis

Data were analysed using Atlas.ti version 23. The relevant documents guided the interpretation of the participants' statements, provided valuable background information and helped contextualise the findings. The data analysis followed the logic of interpretive description, applying Braun and Clarke's (2006) thematic analysis as a systematic approach to identify and interpret patterns in the data (Braun and Clarke 2006). The steps are described linearly for clarity, but in practice the process was iterative, reflexive and interpretive (Braun and Clarke 2006).

Step 1: The interviews and focus groups were conducted, transcribed verbatim and analysed in Dutch from January to November 2024. Preliminary notes were taken on emerging patterns regarding hospitals' approach to the positioning of CANs, which facilitated early interpretive insights. Data collection and analysis occurred in parallel, enabling an iterative and flexible approach.

Step 2: SH and either MH or CvO independently coded the data inductively, developing a consistent coding framework and shared codebook.

Step 3: Initial codes were grouped into subcategories and broader categories that reflected multilevel approaches to position CANs.

Step 4: These categories were refined through repeated cycles of reflexive interpretation into overarching themes capturing the hospitals' multifaceted strategies.

Step 5: Regular meetings ensured consensus on coding, categories and themes. We checked coherence within themes and distinctiveness across themes, repeatedly returning to the raw data to ensure alignment between participants' experiences and our interpretive claims. After analysing 20 interviews and three focus groups, SH created a thematic map to represent the preliminary themes, which the research team reviewed to finalise interpretations. This ensured that the final themes reflected both the descriptive content and the interpretive meanings of the data.

Step 6: The themes were merged into a cohesive narrative that linked the empirical findings to clinically meaningful knowledge, resulting in a detailed explanation of strategies to position CANs. Saturation was reached when no new categories or themes emerged from the data. After 20 interviews and three focus groups, no new themes were identified, and after 23 interviews and five focus groups, no new categories were identified. Field notes further contextualised implementation of strategies. The final themes were translated into English by SH and reviewed by CvO and MH for accuracy.

### 4.6 Ethical Considerations

The study received ethical approval from the Medical Research Ethical Board of Máxima Medical Centre, Veldhoven, the Netherlands (N23.040) on 18 April 2023. Participants were provided with both written and oral information prior to the interviews, which emphasised the voluntary nature of participation and their right to withdraw at any time without consequence. The collected data were stored in a secured folder accessible only to the research team. To ensure anonymity, all data were processed anonymously and individual characteristics were not disclosed. Quotes were presented in a way that prevented linking them to specific individuals.

### 4.7 Rigour and Reflexivity

All authors actively contributed to data analysis, interpretation and manuscript development. Investigator triangulation, involving several researchers in the analysis, ensured diverse perspectives and enhanced the credibility of the findings (Carter 2014). SH is a nursing science PhD candidate with a background in nursing and health and life sciences. She received formal training in qualitative methods and gained experience through research and quality improvement projects. MH is a senior researcher in nursing leadership and the implementation of evidence‐based practices. HV is a professor of nursing science and clinical epidemiologist. CvO is an assistant professor with experience in nursing work organisation and environments. MH and CvO have extensive experience with (analysing) interviews and focus groups. Although all authors were former nurses in academic positions, none had professional ties to the participants, allowing open discussion. The team remained aware of potential bias due to professional proximity and engaged in regular reflection throughout the research process. A scientific advisory board, comprising nursing professors, educators and CANs, was consulted to strengthen reflexivity and reduce bias. Their external perspectives on the study's design, data analysis and interpretation helped to validate and deepen the findings.

## 5 Findings

In total, 23 semi‐structured interviews with relevant stakeholders and five focus groups with CANs were conducted between August 2023 and December 2024. Each interview lasted between 30 and 75 min, with one participant interviewed twice due to time constraints. Each focus group lasted between 80 and 97 min and consisted of 3–5 CANs.

### 5.1 Characteristics of the Participants

Twenty‐one CANs and 23 stakeholders were recruited for the study. Nineteen female participants and four male participants were interviewed (see Table 2) and 18 female and three male participants joined the focus groups (see Table 3).

**TABLE 2:** Interviewee characteristics.

	General hospital	Teaching hospital	UMC	Total
Gender				
Male	1	1	2	4
Female	3	8	8	19
Age (years)				
20–30				
31–40	1			1
41–50	1	5	4	10
> 51	2	4	6	12
Years of experience in current position (years)				
0–5	1	6	5	12
6–10	3	3	5	11
11–15				
Educational level				
BSc		3	1	4
MANP		1		1
Master	3	3	5	11
PhD	1	2	4	7
Role				
CAN		2		2
Board of director			1	1
Nursing director			3	3
Staff advisor	2			2
Manager	2	4		6
Dean		1		1
Senior researcher			2	2
Assistant professor			1	1
Nursing advisory board			1	1
CNIO			1	1
Department manager		1	1	2
HRM advisor		1		1

Abbreviations: BSc, Bachelor of Science; CAN, Clinical Academic Nurse; CNIO, Chief Nursing Information Officer; HRM, Human Resource Management; MANP, Master Advanced Nursing Practice; PhD, Doctor of Philosophy.

**TABLE 3:** Focus group characteristics.

	General hospital	Teaching hospital	UMC	Total
Gender				
Male	1		2	3
Female	3	8	7	18
Age (years)				
20–30		3	6	9
31–40	3	5	2	10
41–50	1		1	2
> 51				
Years of experience in current position (years)				
0–5	3	7	8	18
6–10	1	1	1	3
11–15				
Educational level				
BSc				
MANP	2		1	3
Master	2	8	7	17
PhD			1	1

Abbreviations: BSc, Bachelor of Science; MANP, Master Advanced Nursing Practice; Master, Master's degree; PhD, Doctor of Philosophy.

### 5.2 Themes Representing Positioning of CANs in the Hospital

**FIGURE 1:**
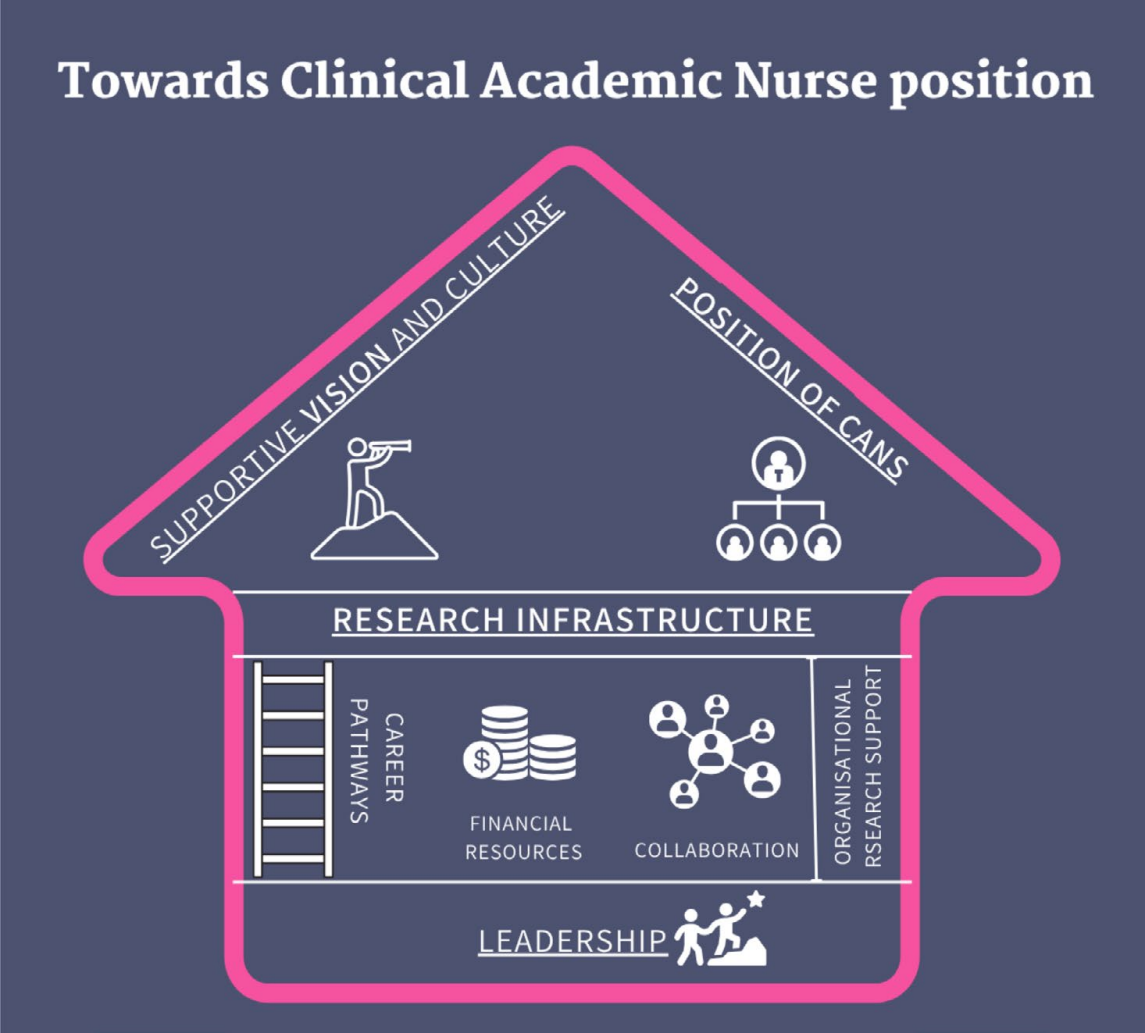
Rich picture of strategies to position CANs.

The hospitals used various strategies to position CANs, spanning different organisational levels from the organisational to the individual level. Four interconnected themes were identified from the thematic analysis: ‘supportive vision and culture’, ‘position of CANs’, ‘research infrastructure’ and ‘leadership’. These themes are visualised in Figure 1, which presents a rich picture from organisational level to individual level, respectively from the roof to the foundation. Per theme, the strategies are displayed in the figure. A strategy was included in the figure if: (1) it is explicitly mentioned as a strategy by participants in the interview, or (2) it has been applied within a hospital.

#### 5.2.1 Supportive Vision and Culture

##### 5.2.1.1 Vision

Some participants indicated that an organisation‐wide vision was needed to position CANs effectively. Across the hospitals included in this study, such visions took on various forms. In one hospital, the academisation of the nursing profession was explicitly included in the vision on nursing care. This vision was translated into a specific profile for CANs, highlighting components such as ambition, the importance of research, integration within the hospital and the roles expected of CANs. Another hospital also formulated a vision focused on the academisation of nurses, but explicitly linked to the strategic goals of the hospital. Although not all participating hospitals had an organisation‐wide vision, participants highlighted the importance of a shared vision on nursing research and the position of CANs to ensure consistent positioning across the hospital.

If the policy is clear, then those managers and department heads will translate it to their department. At the moment that it isn't, they simply won't take action, unless you have very visionary people who are really in favour of innovation and then just make it happen themselves. The so‐called boundary pushers. Dean (20)

In all hospitals, the initial positioning of CANs was driven by committed individuals, such as nursing professors, senior researchers and motivated nurses. In hospitals lacking an overarching vision, it appeared that the positioning relied even more heavily on these individuals, resulting in fragmented efforts based on their personal vision on nursing research. Participants also noted that CANs often leave hospitals without a long‐term vision, due a lack of support for their position and the absence of a defined career path.

Several participants mentioned that it was important to actively involve key stakeholders in the creation of the vision to ensure that these stakeholders understood and committed to the vision. These stakeholders included the board of directors, the Nurse Advisory Board and line management such as team leaders, department heads and managers of a cluster of departments. Participants noted that this collaborative process ensured alignment between the vision and the hospitals' strategic goals and created opportunities for discussions with key stakeholders about the recognition, support and facilitation of CANs. Some participants specifically highlighted the Nurse Advisory Board as an important stakeholder, representing nurses' interests and contributing to strategic decision‐making on the positioning of CANs in the hospital. They indicated that by supporting CANs' interests, the advisory board increased the visibility of CANs among the board of directors and policymakers at the organisational level. Additionally, most participants emphasised that Human Resources (HR) had played an important role in positioning CANs and ensuring its alignment with the agenda of the board of directors. HR, managers and CANs needed to collaborate closely to develop job profiles that support both the needs of CANs and the hospitals' strategic goals.

Participants from various hospitals stressed that positioning CANs required a clear and well‐defined nursing research agenda at the organisational level. They indicated that this agenda ensured that nursing research aligned with the strategic goals and the core values of the hospital and provided direction and visibility to nursing research.

In the meantime, a very strategic framework has been developed with a number of themes, such as digitalisation, better performance of patient outcomes, uh, learning and development (…) a number of goals have been set. These have been discussed with the group of nursing directors and they have embraced this as, this is actually our mission for the years to ensure this. Chair Nurse Advisory Board (19)

Participants from one hospital suggested that it might be important to align the focus of nursing research in the hospital with the research agenda in the medical domain, where clear research priorities had been established.

##### 5.2.1.2 A Culture That Enables Academic Nursing

One nurse director highlighted that a clear vision was needed to drive cultural change. She said that their involvement was important for shaping an organisational culture in which research is integrated into nursing practice. Some participants noted that nursing directors and management acted as equal partners in discussions with the board of directors, physicians and line management because they had mandates to make tactical decisions that aligned with the organisations' vision. This likely enhanced the recognition of nursing research and the CANs' position while ensuring consistent support across the hospital.

then you do need a vision about how you look at the nursing profession, the nursing domain and control over nursing practice (…) If you are convinced of that and with a dose of courage and uh determination yeah, than you can simply come to four CANs in your department. Nurse director (16)

Additionally, the most participants highlighted that the organisational culture was a powerful enabler in realising that vision. They emphasised that a decisive, action‐oriented culture and a culture of continuous learning and improvement promoted the positioning of CANs. They said that such a culture created space for new initiatives and rewarded boldness, which facilitated the positioning of CANs.

##### 5.2.1.3 Clarifying Expectations to Demonstrate Impact

An organisation‐wide vision appeared to harmonise expectations for CANs across all levels of the hospital and to serve as a foundation for translating these expectations to the team level. Most participants emphasised that clearly defined expectations for CANs facilitated the positions' development and evaluation. These expectations included contributing to the development and implementation of scientific knowledge and innovations needed to improve and sustain the quality of nursing care. Participants mentioned that this contribution supported evidence‐based practice and enhanced the hospitals' research profile. Nursing directors, staff advisors and managers emphasised that CANs played an important role in advancing the nursing profession and continuously improving care.

But you will also have to start measuring on the basis of outcomes (…) What the nurse is responsible for and accountable for (…) that lessons are learned from that and that there is a monitoring loop in that (…) Really the professionalisation and continuous improvement of our care. I think that the CAN will have a very important role in that, in that loop. Nurse director (15)

### 5.3 Position of CANs

#### 5.3.1 Clear Job Profiles

To translate expectations into practice, most participants emphasised the importance of a clear job profile for CANs at the organisational level, tailored at team level to suit the local context. They ensured that this approach aligned the organisation‐wide vision and expectations with concrete tasks and responsibilities, enabling CANs to advocate for their role. Participants indicated that a job profile facilitated a uniform and appropriate remuneration structure and enabled CANs to demonstrate their value to line management.

Drafting the job profile was also very valuable in explaining what the added value is of that CAN, (…) also towards the hospital it was important to have that story straight, because you also must get the managers on board, get the doctors on board, and they ask the same thing. They also ask: Well, sounds nice, but what's in it for me, to put it bluntly. CAN (11)

#### 5.3.2 Job Structure as Strategic Instrument

Beyond the job profile, participants from one hospital noted that CANs gained a stronger position through strategic embedding of the nursing domain. They said that this was achieved through a nursing job structure which includes clinical, research and strategic nursing functions like nursing directors, in which the CAN is part of a set of complementary functions such as Chief Nursing Officers (CNO) and Chief Nursing Information Officers (CNIO). The nursing job structure appeared to ensure a visible position for CANs in the hospital and provided a supportive system of stakeholders, including nursing directors, who actively advocated for the CANs' position. These participants emphasised that nursing professors should be appointed within a cluster of departments as strategic research leaders. They mentioned that these professors should serve as mentors and role models for the CANs and help shape the nursing research infrastructure.

Participants from another hospital noted that nursing professors maintain their own research lines while collaborating on several interdisciplinary crosscutting themes, which strengthened their collective work.

Well, and in this way they actually try to strengthen each other, but also to have their own line in which they work very closely with those doctors and also connect with the research institutes. Senior researcher (23)

In another hospital, participants explained that the positions of clinical nurse and nurse researcher were deliberately separated. They felt that the nursing job structure lacked academic career opportunities compared to the scientific job structure which offers a clear pathway, from master's level to full professor. Participants noted that placing CANs within the nursing structure would limit access to these academic pathways. They emphasised that including the academic role of the CAN in the scientific job structure would enable equal interdisciplinary collaboration and increase their academic growth.

Why diminish yourself by really taking on a role as a CAN? Whereas, researcher is just, a, that's just a job of its own. It doesn't really matter what background you come from. Senior researcher (23)

#### 5.3.3 Strategic and Political Drivers in Positioning CANs

The positioning of CANs varied significantly across the hospitals, leading to differences in job titles, influence and authority. These variations appeared to be driven by financial and political considerations. Most participants mentioned that CANs were often funded through departmental budgets, which required managerial approval for their positions. They mentioned that CANs were usually assigned to a department or cluster of departments, which influenced allocation of financial resources. Placing CANs within a single department was costlier than within a cluster, where expenses were shared. Participants from one hospital noted that political considerations influenced the name and definition of the CANs' position to align with managerial priorities. In the focus group of this hospital CANs mentioned that they were designated as ‘master's level nurses’ instead of ‘nurse researcher’, to emphasise their focus on quality improvement. They said that they engaged in discussions with medical and operational managers, where they developed and safeguarded the quality of nursing care, although they were positioned within a clinical ward. Participants from this hospital noted that labelling CANs as ‘master's level nurses’ was a strategic move to highlight their focus on quality improvement.

But if you commit to quality, you know, that is also the responsibility of management. Not research. And, and we did, by choosing quality, we created support. And we also left them with quality alone for a very long time and now we are also taking on the research. And now it makes sense. But if we had started with CANs, the feeling would have been: ‘yes, hands off the bed’. But if we say: ‘quality’. Then it is: ‘yes, support for the head of care and team’. Staff advisors (3 and 4)

Other hospitals used labels like ‘nurse scientist’ or ‘nurse researcher’ for the CANs' position to emphasise that it was one position for the combined role rather than two separate positions.

You are always a CAN. So not hat on, hat off. Some hospitals are going to be very spasmodic, that it becomes a dual job. But it is one function, and you can fulfil that function with different roles. That is how we have chosen, that is what we have chosen very clearly. Eh, and that may mean a little less formation of nurses directly at that, at the bedside. But then it also yields a lot. Nursing director (16)

In these cases, participants noted that the integration of responsibilities into one clearly defined position allowed the salary to be consolidated into one scale, which offered ‘master's level nurses’, ‘nurse scientist’ and ‘nurse researcher’ more attractive remuneration than if the CAN held two different roles, each on separate scales.

In another hospital, participants indicated that in addition to the CANs in departments, some CANs were centrally positioned within a staff department. These central CANs functioned as experts, contributing to the development of nursing research policies and providing operational support to CANs, such as mentorship. Participants also suggested that appointing a CAN as part of a tripartite management structure, alongside the business and medical manager, could strengthen the alignment between nursing research and strategic goals within clinical clusters.

So I see an important role for the tripartite CAN. Because how wonderful would it be to immediately put that nursing research question in that line management? You have to demonstrate that you can improve care, the quality of care… well, in general they also want to see what the improvement is. Well, then you can immediately link your research question to the improvement goals, or set out your research. CAN (11)

### 5.4 Research Infrastructure

Alongside a clear and strategically embedded position, almost all participants emphasised that a well‐developed research infrastructure was needed for the positioning of the CANs. Some participants noted that CANs benefited from participation in existing nursing research programs and support systems, rather than building research structures from scratch. These support systems included tailored organisational research support such as connecting with local science departments, strategic financial resources, including grants and departmental budgets to finance CANs' research time, interprofessional research collaboration with peers, physicians and educational institutions, as well as structured academic career pathways.

#### 5.4.1 Tailored Organisational Research Support

Participants noted that it was important to connect CANs with local scientific departments in the hospital to support them in their position and professional development. These departments appeared to develop research policies and supported CANs in their research. Furthermore, these departments seemed to offer access to specific expertise and training, including methodological guidance and software tools tailored to nursing research.

You have a few PhD doctors in this hospital who can support you to a certain extent (…). But I have to figure everything out myself. And that, for that, I really need the academy. (…) Access to PubMed, SPSS on your computer. CANs focus group (28 and 29)

#### 5.4.2 Strategic Financial Resourcing

Most participants observed a tension between patient care budgets and financial resources of nursing research. Without dedicated research grants, CANs often relied on departmental budgets and managerial approval to allocate research time. The participants noted that grants were important in order to give CANs time for research alongside patient care. However, CANs often struggled to obtain research funding due to limited experience with grant applications. Participants from one hospital noted that nursing professors and senior researchers supported CANs in grant acquisition. Additionally, a nursing director applied a strategic grant approach by involving grant advisors and financial controllers to support writing and budgeting. Instead of focusing on one large grant, they diversified funding by applying for multiple smaller grants.

But we are also looking at, can't we just get money from multiple parties that we all ask to put 20 of 25 of 50,000 euros in, so that you might get 4 of the 6 parties that still get 4 years of profit. (…) more small pots uh can sometimes be a bit easier, if you want the amount of ZonMw applications and KWF subsidies that are available, (…) that's not that much either. Uh plus you uh there are a number of more privateers on the coast. Nursing director (20)

Several participants noted that the use of departmental budgets and internal and external grants provided greater flexibility and ensured research time for CANs. Grants appeared to play an important role in the support for career development for nurses who wanted to develop in research. The participants from all hospitals noted that to increase the number of masters'‐prepared nurses, hospitals funded masters' degree programs for the CANs through grants. Moreover, one hospital proposed a support plan for academic pathways for nurses, including funding for pre‐PhD, PhD and postdoctoral fellowships. Participants from that hospital noted that the program aimed to establish a sustainable nursing research infrastructure, increasing scientific publications, fundraising capacity and the impact of research for patient populations and the nursing profession.

#### 5.4.3 Interprofessional Collaboration

Almost all participants highlighted the importance of a strong internal peer network for CANs, with various experience levels that support informal sparring, peer‐to‐peer learning and mutual empowerment. CANs mentioned that an external peer network offered fresh perspectives, new energy and collaboration beyond the organisation.

Several participants suggested the importance of collaboration between CANs and physicians for sharing research experience and successful grant writing. Their involvement in nursing research could enhance its impact and could create a stronger connection to the medical staff board, thereby fostering broader support for nursing research initiatives.

Some participants suggested that strategic alliances between hospitals and research institutes, such as universities and universities of applied sciences, could be important in formulating joint research themes. These themes could also extend beyond the hospital environment to other healthcare institutions, such as nursing homes and home care.

Because of course, as an academic setting, we don't just have to organise things for ourselves; you actually also want to be able to position a kind of nursing science within your care programs, care pathways, across the boundaries of your own department (…) in your region, in home care, or possibly even in the nursing home sector. Nursing director (16)

Participants suggested that these collaborations could strengthen the position of nursing research within hospitals, by better aligning it with broader academic agendas and thereby increasing its visibility and credibility. Some participants mentioned that they could facilitate access to more funding opportunities and enable alignment with regional research priorities, which contributes to the development of regional and even national knowledge networks. One participant said that these collaborations could also create synergy between education and clinical practice, enhancing research capacity and providing career opportunities for CANs. Moreover, she said they could enable nursing professorships or academic chairs and mentorship for CANs, particularly in hospitals lacking these support structures.

#### 5.4.4 Academic Career Pathways

Almost all participants indicated that a structured academic career pathway, including positions for nurses with master's degrees, PhDs, postdoctoral fellows, principal investigators and professors, could be supportive in creating a solid infrastructure for nursing research. Such a career path could reduce the reliance on individuals, including line management to facilitate the position and career opportunities of CANs.

We just want it to be rock solid and not so dependent on those people. And if we move towards that in every division there is such an infrastructure with master‐trained nurses, with PhD candidates, with postdocs and possibly a lecturer UHD or professor. Then you will see that they also have the ability to raise money themselves, then you are no longer so dependent on people around you. Senior researcher (23)

One senior researcher suggested that academic career pathways for CANs could be modelled after those of physicians by integrating the ‘trias academica’. ‘Trias academica’ brings together research, teaching and clinical practice in the nursing job structure, which supports the professional development of CANs. Moreover, participants noted that career prospects for the CANs also lay in professionalising the CANs position, including pursuing doctorates and thereby advancing research expertise. Most participants noted the absence of formal positions for doctoral CANs within their hospitals. They suggested that doctoral CANs could explore leadership positions such as research group leaders, program directors or staff and line management positions.

### 5.5 Leadership

Besides the components of the research infrastructure, several participants indicated that the CANs actively engaged in defining their role within their hospital. They described taking initiatives to align their research focus with both the needs of their department and the broader strategic goals of the hospital. Staff advisors noted that the CANs increased their visibility and communicated the outcomes of their work to key stakeholders. They mentioned that CANs translated research findings into practical information and engaged in dialogue with nursing colleagues and line management.

They do presentations, they profile themselves well and everyone now sees the added value. Also the heads of care, who like having a CAN. Someone who can think and help along at a level. So it works very well. Staff advisors (3 and 4)

Additionally, some participants indicated that CANs participated in symposia and research meetings to increase their visibility within the hospital. Participants mentioned that the CANs contributed to research‐related activities by involving nursing colleagues in research projects, providing support during research processes and sharing their knowledge and expertise during training sessions.

However, some participants indicated also that the CANs were pioneers in positioning themselves, as the necessary infrastructure was still insufficiently developed in some hospitals. This meant that the CANs were often responsible for positioning their own role in a context with limited support. Participants noted that this lack of guidance and resources led to feelings of uncertainty and a sense of being left to navigate their role independently when they first started in their position.

Almost all participants highlighted that since the CAN position was not yet fully embedded in many hospitals, key stakeholders such as line managers were often unaware of its added value and necessity. Convincing these stakeholders required strong leadership skills. Participants suggested that CANs should define clear outcome measures, together with these stakeholders, to demonstrate their impact. However, several participants noted that the CANs were often relatively junior in their position and might lack the strategic insights and political sensitivity needed to effectively negotiate about their position, responsibilities and professional and personal development.

R1 You have to prune the bushes yourself time and time again, to make sure that you clear that path again.

R4 And keep reflecting on yourself and am I doing this right? What should I take with me in this? What should I do differently next time? So yes, that, that role of pioneering, that is, I think, also part of the CAN or Advanced Nurse Practitioner who do research. (…) Yes, you really have to have something of that, because no one is going to hand it to you. CANs focus group (28 and 29)

Most participants indicated that CANs were encouraged to actively network to shape their position and expand research opportunities. They noted that networking played a particularly important role during the pioneering phase of the function. It helped CANs identify and engage key decision makers who have the mandate to influence strategic decisions and strengthen the position of the CANs, such as policy advisors, the Nursing Advisory Board, the CNO, CNIO, research coordinators and line managers. Additionally, the CANs mentioned that they established contacts with nursing professors, senior researchers and nursing directors outside their hospital for mentoring and collaboration in research projects to enhance their research skills. CANs also emphasised the importance of networking with peers to avoid the sense of ‘pioneering alone’, which often arises in the absence of seniority. CANs saw networking as a way to increase mutual support, reduce professional isolation and strengthen the position of CANs by encouraging shared learning and collective visibility.

## 6 Discussion

This study identified empirically grounded strategies to position CANs within Dutch hospitals. The findings revealed four interrelated themes in which the strategies are embedded involving a clear vision, a supportive organisational culture, a strong research infrastructure and CANs' leadership. Together, these findings will provide practice‐based knowledge to position CANs in hospitals. These themes are mutually reinforcing. Organisational vision sets the direction and organisational leaders translate this into roles and resources. Research infrastructures provide the practical means for CANs to conduct research, while leadership of CANs ensures the capacity and influence to enact these ambitions in practice. It is important to note that some aspects mentioned by participants were observed in only one hospital, yet they may hold relevance for others. This variation reflects the differing stages of development of CANs and their roles across hospitals.

### 6.1 Organisational Vision and Culture

This study shows that an organisational vision requires more than top‐down policy. It demands active involvement from strategic stakeholders like boards of directors, line management and the Nurse Advisory Board. Also, Paterson et al. emphasised that organisational managers must articulate a vision for clinical academic nursing roles and their impact (Paterson and Strickland 2023). This requires a shift moving away from short‐term, productivity‐driven approaches towards long‐term strategies focused on quality and innovation. Therefore, organisational leaders should show courage and commitment, as they are key gatekeepers in the positioning of CANs (Aspinall et al. 2024). Yet, limited research literacy among managers may hinder progress, underscoring the need for education on nursing research (Aspinall et al. 2024). Active engagement of managers is important as this study indicated that in some hospitals, nursing directors and managers functioned as cultural agents, serving as key stakeholders in facilitating nursing research and positioning CANs in alignment with the organisations' vision and culture. Rickard et al. also argued that an organisational culture supportive of research is essential for positioning CANs effectively (Rickard et al. 2011). Therefore, improvement outcomes that demonstrate the impact of CANs should be targeted to assist organisational leaders in making informed decisions (Granger et al. 2022), enabling them to subsequently become cultural agents.

### 6.2 Demonstrating Impact of CANs

This study showed that CANs should define clear outcome measures with key stakeholders to demonstrate their impact. While the contributions of CANs in advancing evidence‐based practice, improving patient care and fostering organisational development through research are increasingly acknowledged, recognition remains inconsistent across settings (Newington et al. 2021). This may be due to the still‐developing status of nursing as an academic discipline (Barrow 2023), which limits visibility of CANs' impact at organisational levels. This results in variation in institutional awareness and leadership commitment to the positioning of CANs, leading to differences in how CANs are supported and positioned across hospitals, as confirmed by this study. Demonstrating the impact of CANs in measurable performance terms can enhance their visibility, raise stakeholder awareness and help reduce variation in their positioning by promoting stronger and more consistent organisational support (Aspinall et al. 2024). Studies by Ede et al. and Coad et al. showed that CANs improved care processes, data use, staff engagement and service innovation (Coad et al. 2019; Ede et al. 2025). Some of these impacts were quantified in quality indicators such as funding, publications and retention (Coad et al. 2019). Hence, making the impact of CANs visible is important for strengthening the nursing profession, for reinforcing the position of CANs and for ensuring tangible benefits for patients, staff and healthcare organisations. To translate this impact into sustainable practice, adequate financial and structural support is essential (Coad et al. 2019).

### 6.3 Financial Resourcing and Formal Positions

This study showed that CANs often rely on departmental budgets and grants and that combining these resources can lead to structural financial support. Johnson et al. emphasised protected research time and structural funding as key to developing clinical academic career pathways in nursing (Johnson et al. 2024). Moreover, prioritising nursing research at the organisational level enables the allocation of structural financial resources for CANs (Paterson and Strickland 2023), potentially creating a positive vicious cycle. With secured funding, CANs could be less dependent on temporary project‐based grants, which may foster continuity and support the consistent production of compelling evidence regarding their impact. Furthermore, this study highlights the importance of formal positions and clear job profiles for CANs. When CANs have formal positions and institutional support, they can grow into senior research positions and role models, both important for strengthening the research infrastructure (Coates and Mickan 2020). A uniform and stable positioning of CANs also reduces the risk of them focusing on only one aspect of their hybrid role, clinical or research, improving their retention within the organisation (Ede et al. 2025).

### 6.4 Leadership Competencies and Strategic Alliances

In addition to secured funding, this study found that CANs need leadership competencies such as political awareness and networking to profile their position and engage key stakeholders. Similarly, Van Dongen et al. found that PhD‐prepared nurses needed further preparation in leadership competencies, team leadership and research project management (van Dongen et al. 2025). Also, mentorship and support from senior researchers support CANs' confidence, career advancement (van Dongen and Hafsteinsdottir 2022) and support endorsing structured clinician‐researcher pathways (Johnson et al. 2023). In this study, senior researchers were found to play a pivotal role in building partnerships between hospitals and educational institutions, thereby aligning research agendas and expanding knowledge networks. As Henshall et al. emphasise, these partnerships are most effective when supported by shared leadership, transparent communication, and well‐aligned institutional infrastructures (Henshall et al. 2021).

Furthermore, collaboration with academia can also facilitate the establishment of endowed chairs, which recognise the value of nursing science, enhance researcher credibility, and strengthen research infrastructure (Randhawa et al. 2014). Although not derived directly from the findings, the research team viewed this as a relevant insight from the broader literature. While general nursing science chairs provide an important disciplinary foundation, specialised chairs align more closely with clinical practice (Wallis and Chaboyer 2012), fostering domain‐specific expertise, interprofessional collaboration and the translation of research into patient care.

### 6.5 Strengths and Limitations of the Work

A strength of the study is the inclusion of various hospitals and stakeholders, providing a broad perspective on CAN positioning and enhancing generalisability. Data triangulation through focus groups, interviews and documents further strengthened validity and reliability, with documents offering valuable contextual insights for a more nuanced understanding of the research context. However, the inclusion of only current CANs limited understanding of role discontinuation. Also, the focus on hospitals with advanced CAN positioning enabled in‐depth exploration of strategies but may underrepresent challenges in less developed settings.

### 6.6 Recommendations for Further Research

To strengthen the position and impact of CANs, research should assess their contributions to nursing practice, patient outcomes, personnel well‐being and organisational performance. Such studies will build an evidence base for the structural positioning of CANs and for the growth of nursing as an academic discipline. Studies on CANs' leadership development and career trajectories can enhance role sustainability and retention. Furthermore, comparative studies across different healthcare organisations and countries may offer valuable insights into effective global strategies for positioning CANs.

### 6.7 Implications for Policy and Practice

Positioning CANs requires courageous, forward‐thinking leadership at both strategic and operational levels. Boards of directors and line managers should move beyond short‐term productivity goals and adopt long‐term strategies that prioritise quality, innovation, and the integration of research into nursing practice. This demands an organisation‐wide vision, institutional commitment and a sustainable research infrastructure. A well‐integrated Nurse Advisory Board can help align the positioning, support and evaluation of CANs with policy priorities.

Strategic partnerships between hospitals and research institutions should be actively developed to align research, policy, and education through joint research agendas. These alliances should formalise professorships or lector positions and foster structured mentorship and strengthen grant acquisition capacity.

Once positioned, CANs' contributions should be demonstrated through measurable goals and systematic evaluation of outcomes. By proactively aligning their research with clinical and organisational priorities, CANs can maximise their impact. Showcasing their expertise through innovation projects, dissemination of results to practice and organising symposia increases visibility, drives implementation and advances the nursing profession.

## 7 Conclusion

This study highlights the key strategies to position CANs in Dutch hospitals, including a clear vision, defined positions for CANs, a strong research infrastructure and leadership of CANs. Limited institutional commitment creates a vicious cycle: without support, CANs cannot be effectively positioned, limiting their impact on outcomes, which further reinforces the lack of commitment. To break this cycle, hospitals must adopt long‐term strategies that integrate research into clinical practice and recognise CANs as strategic assets. Formalised strategic collaboration between hospitals and research institutions is important to develop clinical academic pathways that align policy, education, research and practice in health care.

## Funding

This work was supported by ZonMw, the Netherlands Organisation for Health Research and Development. Grant number: 10040022220039.

## Ethics Statement

The study received ethical approval from the Medical Research Ethical Board of Máxima Medical Centre, Veldhoven, the Netherlands (N23.040).

## Conflicts of Interest

The authors declare no conflicts of interest.

## Data Availability

The data that support the findings of this study are available from the corresponding author upon reasonable request.
